# Awareness of preventive medication among women at high risk for breast cancer and their willingness to consider transdermal or oral tamoxifen: a focus group study

**DOI:** 10.1186/s12885-015-1893-6

**Published:** 2015-11-09

**Authors:** Lindsey C. Karavites, Subhashini Allu, Seema A. Khan, Karen Kaiser

**Affiliations:** Department of Surgery, University of Illinois College of Medicine at Mt. Sinai Hospital, 1500 South Fairfield Avenue, Chicago, IL 60608 USA; Department of Surgery, Northwestern University Feinberg School of Medicine, 250 East Superior Street, Suite 4-420, Chicago, IL 60611 USA; Department of Surgery, Northwestern University Feinberg School of Medicine, Prentice Women’s Hospital, 250 East Superior Street, Suite 4-420, Chicago, IL 60611 USA; Department of Medical Social Sciences, Northwestern University Feinberg School of Medicine, 625 North Michigan Avenue, Suite 2700, Chicago, IL 60611 USA

**Keywords:** Breast Tumors, Preventive medicine, Local transdermal therapy, Focus group, Tamoxifen

## Abstract

**Background:**

Despite demonstrated efficacy, acceptance of selective estrogen receptor modulators (SERMs), such as tamoxifen, for breast cancer risk reduction remains low. Delivering SERMs via local transdermal therapy (LTT) could significantly reduce systemic effects and therefore may increase acceptance. We aim to assess women’s knowledge of breast cancer prevention medications and views on LTT of SERMs.

**Methods:**

Focus groups were conducted with healthy women identified through the comprehensive breast center of a large urban cancer institution. Group discussions covered risk perceptions, knowledge of and concerns about risk reducing medications. Participants reported their perceived risk for breast cancer (average, below/above average), preference for SERMs in a pill or gel form, risk factors, and prior physician recommendations regarding risk-reducing medicines. Participants’ breast cancer risk was estimated using tools based on the Gail Model. Trained personnel examined all qualitative results systematically; risk perceptions and preferred method of medication delivery were tallied quantitatively.

**Results:**

Four focus groups (*N* = 32) were conducted. Most participants had at least a college degree (78.2 %) and were of European (50 %) or African ancestry (31 %). The majority (72 %) were at elevated risk for breast cancer; approximately half of these women perceived themselves to be at elevated risk. Few participants had prior knowledge of preventive medications. The women noted a number of concerns about LTT, including dosage, impact on day-to-day life, and side effects; nonetheless, over 90 % of the women stated they would prefer LTT to a pill.

**Conclusion:**

Awareness of preventive medications was low even in a highly educated sample of high-risk women. If given a choice in the route of administration, most women preferred a gel to a pill, anticipating fewer side effects. Future work should focus on demonstrating equivalent efficacy and reduced toxicity of topical over oral medications and on raising awareness of chemopreventive options for breast cancer.

## Background

Breast cancer is the most common cancer in women in the United States, with more than 232,000 cases of invasive cancer diagnosed in 2014 [1]. It is the second leading cause of cancer-related deaths among US women, claiming over 40,000 lives each year [1]. Although mammographic screening and advances in the treatment of breast cancer have increased survival rates, primary prevention of breast cancer remains a high priority since it will allow women to avoid the trauma and toxicity of cancer therapy, and eventually may also decrease breast cancer death rates [1].

Selective estrogen receptor modulators (SERMs) prescribed as preventive medications hold promise of reducing the breast cancer burden. Tamoxifen, used for both pre- and postmenopausal women, and a second orally administered SERM, raloxifene, used in postmenopausal women, are the only medications currently approved by the FDA for use in the United States in the prevention of breast cancer in women at increased risk for the disease [2]. In the Breast Cancer Prevention Trial (BCPT), tamoxifen was shown to halve the incidence of invasive breast cancer among high-risk women, and demonstrated an even greater reduction in incidence of the disease among high-risk women with cellular atypia [3]. However, despite its well-documented benefits, tamoxifen is associated with a number of risks and side effects, including menopausal symptoms (hot flashes), venous thrombo-embolism, cataracts, and increased risk for endometrial cancer [4–6]. Because of these adverse effects, tamoxifen is accepted by only 29–42 % of the women offered the drug for chemoprevention [4, 7]. Moreover, several studies have shown that the highest rates of noncompliance are in younger women who would stand to benefit the most from the medicine [8–10]. In addition, among women who accept tamoxifen for chemoprevention, 49 % interrupt or abandon therapy due to side effects or fear of side effects [11]. Further, high-risk women who are candidates for chemoprevention have indicated reluctance to take a pill daily for five years, which is the current mode of administration and recommended duration of tamoxifen therapy [12, 13].

Delivering tamoxifen directly to the breast tissue via the skin, i.e., local transdermal therapy (LTT), is a novel approach with anticipated advantages of reducing or possibly eliminating systemic toxicities associated with oral therapies and avoiding individual variations in drug metabolism that could alter drug effectiveness. A small phase II study demonstrated that an active metabolite of tamoxifen, 4-Hydroxytamoxifen (4-OHT), applied to the skin of the breast as a gel, reduced breast tumor cell proliferation to the same extent as oral tamoxifen [14, 15]. We have conducted two additional clinical studies to further test the success of transdermal drug delivery to the breast. In one study, we compared transdermal 4-OHT to oral tamoxifen in a window trial of 26 women with ductal carcinoma in situ (DCIS). Breast concentrations of 4-OHT in the oral and transdermal groups were similar, with equivalent suppression of cell proliferation (measured as Ki-67 labeling) suggesting equivalent efficacy in DCIS lesions, while the plasma concentration of 4-OHT was 5-fold lower when compared to the oral tamoxifen group [16]. This study confirmed that the gel formulation of an active tamoxifen metabolite has the potential to perform as well as the oral form of the medication with little systemic exposure. In our second study, we randomized 30 women undergoing mastectomy to a diclofenac patch applied either to the breast skin or the abdominal skin. Results indicated significantly higher concentrations of diclofenac in breast tissue in those applying the patch directly to their breast (manuscript accepted for publication). These results showed that drugs applied to the breast skin will preferentially concentrate in the parenchyma to a greater degree than if applied to skin elsewhere, and that biomarker endpoints suggest efficacy.

From this early experience, we conclude that topical application of 4-OHT offers promise as an effective preventive medication that women may find more acceptable than oral medication. As we continue efforts to develop LTT for breast cancer prevention, we must establish that these efforts will actually translate into increased acceptance of this method of drug delivery among at risk women. Therefore, we examined women’s perceptions of preventive medicines and the acceptability of a topical application of this active metabolite of tamoxifen among a sample of high risk and average risk women without a prior breast cancer diagnosis.

## Methods

### Eligibility

Because views of preventive medications may vary by a women’s risk of developing breast cancer, we included women at average and elevated risk of breast cancer. For recruitment purposes, elevated risk was defined as risk greater than that of the average-risk peer of the same age or by virtue of possessing one or more of the following conditions: a) a 1st or 2nd degree relative with breast cancer diagnosed at any age; b) multiple prior breast biopsies regardless of histology; c) prior or current evidence of breast epithelial atypia (histologic or cytologic); d) estimated mammographic density on visual inspection of BIRADS 3 or 4; e) known carrier of BRCA 1 or 2 mutation; and/or f) prior history of chest wall radiation. Women were considered average risk if they possessed no more than one of the following characteristics: a) no family history of breast cancer in a first or second degree relative; b) one prior breast biopsy without atypia, and c) BIRADS 1 or 2 estimated mammographic density on visual inspection. All participants were female age 21 or older.

### Recruitment

Participants were identified through the Bluhm Family Program for Breast Cancer Early Detection and Prevention at Prentice Women’s Hospital, Lynn Sage Comprehensive Breast Center. Eligible women received a phone call from the study coordinator inviting them to participate in a focus group about women’s views of their breast cancer risk and methods to prevent breast cancer.

### Focus groups

Focus groups were held in Prentice Women’s Hospital, part of the Chicago campus of the Feinberg School of Medicine of Northwestern University. An experienced focus group moderator led consented participants through a discussion of breast cancer risk perceptions and knowledge of and concerns about risk reduction medications. Each woman was asked to characterize her own risk as below average, average, or above average as part of the discussion. After discussing knowledge and concerns of risk reduction medications, the moderator provided a brief overview of LTT; the women then discussed their views of the acceptability of a skin application of risk reduction medication. Focus group sessions lasted approximately 60 min. The discussions were audiotaped and transcribed. At the conclusion of the group, each participant completed a short survey to provide sociodemographic information, breast cancer risk factors (i.e., prior DCIS or LCIS diagnosis, menstrual and pregnancy history, family history of breast cancer, number of prior breast biopsies, and prior breast biopsies with atypical hyperplasia), and whether a physician had ever suggested they take medication to reduce their breast cancer risk. At the completion of the group, participants were given a handout providing information on the two FDA approved medications currently in use for breast cancer prevention. The moderator provided information to participants at the end of the focus group discussion, which addressed their questions. These included the instructions for application, odor, and consistency. Participants received $50 compensation for their time and a voucher to cover parking expenses in a Northwestern University parking structure. Following completion of the focus groups, participants’ medical histories (biopsy history, BRCA status, breast density, and recommendations to take preventive medications) were obtained via medical record review. The Northwestern University Internal Review Board approved all procedures.

#### Analysis

Focus group transcripts were content analyzed by the first and last author. First, the number of women in each group with prior knowledge of risk reduction medications was tallied. Next, thematic analysis identified concerns about preventive medicines and concerns about a topical preventive medication. Concerns were identified and tallied within and across groups. The analysts compared their results and any differences were discussed and resolved. Each participant’s breast cancer risk was calculated using the Breast Cancer Risk Assessment Tool provided by the National Cancer Institute and the Detailed Breast Cancer Risk Calculator created by Halls, both are based on the Gail Model. Participants were classified as below, at, or above the average risk for women of their age [17, 18]. Participant survey responses and their perceived risk were tallied.

## Results

Four focus groups were conducted with 32 women. A summary of participant sociodemographic characteristics, breast cancer risk factors, and knowledge of preventive medications is shown in Table 1. The majority of the sample was of European (50 %) or African ancestry (31 %). Women ranged in age from 25 to 67 years. Despite the high educational attainment of the group (94 % of the participants attended college), less than 20% of the women had any prior knowledge of preventive medicines for breast cancer. Based upon our recruitment criteria, 20 of the women were classified as high risk; 12 were classified as average risk. Table 1 shows the women’s estimated breast cancer risk according to the Gail Model for women over age 35 and Halls’ Model for those younger than 35 (*N* = 4). Twenty-three women were above peer average, 3 were at the peer average, and 6 were below peer average.

**Table 1:** Focus Group participant demographics, breast cancer risk, *N* = 32

Characteristic	
Mean Age	48 (25–67)
Education	
Some High School	2 (6 %)
Some College	5 (16 %)
College Degree	11 (34 %)
Graduate Degree	14 (44 %)
Ancestry	
European	16 (50 %)
African	10 (31 %)
Other	6 (19 %)
Ethnicity	
Hispanic	4 (13 %)
Non-Hispanic	28 (87 %)
Mean Age of Menarche	12 (9–17)
Mean Age at 1^st^ Live Birth	29 (16–39)
1^st^ or 2^nd^ Degree Female Relative with Breast Cancer	21 (66 %)
History of Breast Biopsy	16 (50 %)
History of Biopsy with Atypia	3 (9 %)
BRCA 1/2 Mutation Carrier	0 (0 %)
BIRADS Mammographic Density	
0 – No Mammogram	3 (9 %)
1 – Fatty	1 (3 %)
2 – Scattered Fibroglandular	9 (28 %)
3 – Heterogeneously Dense	19 (60 %)
4 – Extremely Dense	0 (0 %)
Prior Knowledge of Preventive Medicines	6 (19 %)
MD Recommended Preventive Medicine	2 (6 %)
Estimated Breast Cancer Risk (Gail Model)	
Above Peer Average	23 (72 %)
At Peer Average	3 (9 %)
Below Peer Average	6 (19 %)

### Perceived risk of breast cancer

**Fig. 1:**
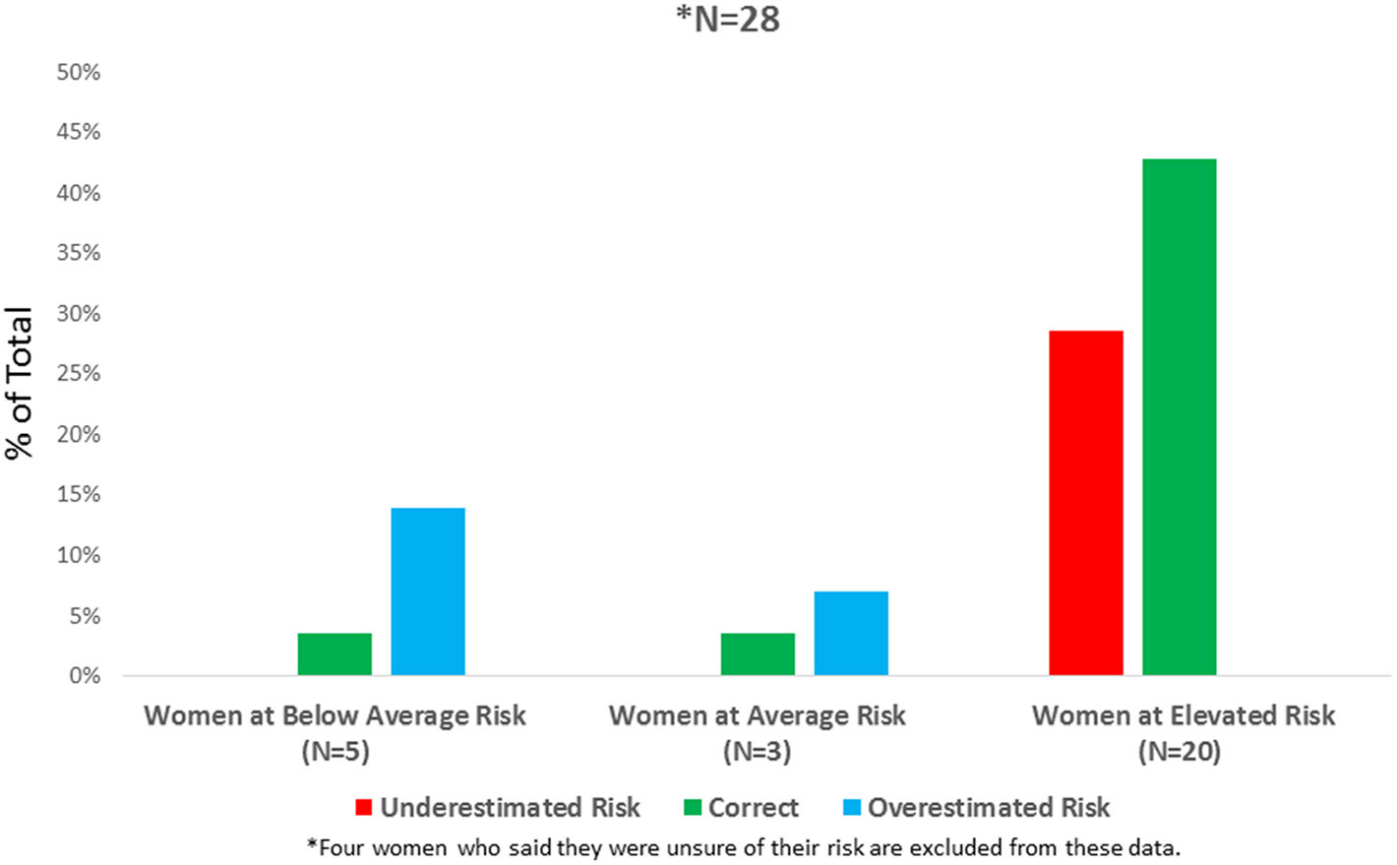
Perceived vs. Calculated Risk, Focus Group Participants, *N* = 28. The participants’ breast cancer risk was estimated using assessment calculators based on the Gail Model. They were grouped according to their risk relative to that of their peer group as below, at, or elevated risk. 5 were calculated to be below average risk. Among these women, 14 % of all participants, represented in blue, overestimated their risk, while 3.6 % correctly perceived their risk as below average, represented in green. For the women calculated at peer average, 7 % overestimated their risk and 3.6 % correctly identified their own risk, again shown in green. No one in this group underestimated their risk. Finally, in the high-risk group of 20 women, 43 % correctly perceived themselves as high risk, green, while 29 % underestimated their risk, red. The total number of women that overestimated their risk was 21 %, while 29 % underestimated their risk and 50 % correctly identified their own risk. 4 women who were unsure of their risk were excluded from these data

When comparing women’s perceived breast cancer risk with Gail Model estimates, women correctly assessed their own personal risk 50 % of the time (Fig. 1). Women overestimated their risk 21 % of the time and underestimated their risk 29 % of the time. Notably, over one third of women at increased risk for breast cancer perceived themselves as average or below average risk. One high-risk woman stated, “I would say (my risk) is average ‘cause I choose to be positive. My sister passed from breast cancer…which kind of made me a little, feel, a little high risk…but my mother…she beat it…I’m hopeful, though.” Another high-risk woman said, “I consider myself low risk because none of the women in my family have ever had breast cancer, so I feel fortunate and not that worried.”

When asked what drove their risk perceptions, women most often cited family history. For example, one woman who perceived herself as above average yet was calculated to be at average risk stated, “I think I have a greater risk because of a couple of factors, number one is family history of breast cancer.” Other factors that shaped women’s perceived risk included obesity, poor diet, smoking, stress, environmental pollution/toxins, hormone replacement therapy, nulliparity, breast augmentation, abnormal mammogram, dense breast tissue, history of breast biopsy and genetic mutations.

### Knowledge of preventive medications

Very few of the focus group participants were aware that medications existed to prevent breast cancer. One woman mentioned the drug Evista, stating that she knew “some people use it for osteoporosis and they say it helps reduce your risk for breast cancer, too.” Another woman admitted that she had heard of tamoxifen but when asked what she knew about the medication she stated, “There are some not great side effects…I mean, it sounds a lot like menopause.” Others who knew of tamoxifen were not aware of its application to disease prevention and thought it was only indicated for treatment of disease. “We hear a lot about what’s going on in the research world and tamoxifen…and just that it’s out there, but I don’t know anything, specifics about reduction of rates, or side effects, or anything like that.” Even those familiar with the medication had limited knowledge of its purpose. By comparing women’s focus groups comments about preventive medications with their risk perceptions, we ascertained that participants who perceived themselves to be at high risk for breast cancer tended to be more knowledgeable of preventive medications than those who did not perceive themselves to be at elevated risk, 38 % vs 0 % respectively. Moreover, women with awareness of preventive medications tended to be older and had either a first-degree relative with the disease or had undergone a breast biopsy.

### Concerns about preventive medications

When prompted to list concerns they would have if their doctor recommended that they take preventive medicine, women in every group wanted to know about side effects of the medicine. Further, women stated that they would want their physician to give them an individualized risk/benefit analysis and demonstrate that they would see a significant risk reduction on the drug. A few women voiced reluctance to the idea of taking medicine for any indication and claimed that it would take a lot of convincing and evidence provided to them by their physician before they would consider accepting a drug. Duration of therapy, interactions with other medications or products, cost, and whether medicines used for prevention would be covered by insurance were other major concerns. Also, several women noted fears of taking pills, and in some cases, difficulty swallowing them. The following comments are representative of their concerns:

“I think the side effects are traumatizing relative to what you’re treating. I mean just like incontinence and like all this other stuff that comes up (from taking various medications) that’s not even related to what it’s treating…so you kind of wonder, why? Why would you want to take this thing that could create these six other problems?”

“Anything I have to take for something that I am treating, I can deal with what I have to deal with, but just as far as on the level of prophylactics, I would want it to be pretty simple that way.”

“I’d like to know the risk benefit analysis on an individual basis, what is the risk of not taking the medicine versus the risk of taking the medicine. So how elevated would my risk be for breast cancer if I didn’t take it, and what are the benefits if I did take it, and then look at the side effects.”

“I think probably the cost as well, I mean, don’t want to send everybody to poor house… or myself trying to prevent something”

“I’ve never been a pill person, so I’ve never wanted to, I wouldn’t want to start anything that I’d have to continue”

“I think that apart from the side effects, I would also want to know the ease of taking the medicine, because I hate taking pills and I gag and choke even when it’s a tiny pill.”

### Acceptability of skin application of tamoxifen

Although women had a number of reservations about preventive medications, they were more open to the idea of a gel application of the medicine. Over 90% of focus group participants stated they would prefer the skin application of preventive medicine over a pill application if a physician advised them to take preventive medication (Table 2). Of the remaining individuals, 6% were unsure of which version they would prefer and would need more information to make their decision, while one subject stated that she would prefer to take a pill. The most common reason given for preferring a skin application was a desire to avoid systemic side effects from a pill. Other common reasons included a dislike of taking pills and a preference for administering medication directly to the site where it is needed. There was no correlation between women’s risk perceptions and their preference for or against the gel.

**Table 2:** Focus Group participant willingness to use skin application of drug, *N* (%)

Which would you prefer for breast cancer prevention if you were offered a choice between a pill and a gel?	^a^Perceived below average and average risk	Perceived above average risk	Unsure of personal risk	All participants
Gel	11 (34 %)	15 (47 %)	3 (9 %)	29 (91 %)
Pill	0 (0 %)	1 (3 %)	0 (0 %)	1 (3 %)
Unsure	1 (3 %)	0 (0 %)	1 (3 %)	2 (6 %)
Total	12	16	4	32

^a^Only two women perceived themselves as below average in risk of developing breast cancer and were therefore combined with those who perceived themselves as average to simply the table

### Concerns about skin application of tamoxifen

Focus group participants mentioned a number of concerns they would want to discuss with their physician prior to taking a topical preventive medication, including the impact of the gel on day to day life, dosage, characteristics of the gel, side effects, and whether the gel posed a threat to others. Women in every group questioned whether they could continue with normal activities (e.g., bathing, exercising) while using the gel. They were also concerned about the gel staining their clothes, whether they would have to wait to get dressed after applying the gel, and how soon after application they could bathe.

The women raised questions about the dosage of the gel: How long would they take it? How much gel would they apply each day? Does it need to be applied at the same time each day? They questioned whether a gel could be dosed properly, particularly given varying breast sizes among women. In addition, they wanted to know more about the characteristics of the gel, including its texture, scent, packaging, and storage requirements.

Side effects were also a concern. The women wondered about possible negative effects on hands, impact on organs near the application site (e.g., lungs, heart), and changes to the texture of the breast skin. One woman remarked, “I would look at those side effects really, really carefully and probably consult other doctors who could either put my mind at rest or convince me not to take it.” A 45-year-old woman who perceived her risk of breast cancer to be above average said, “I mean I don’t know what the risks are, but I would want to know the quality of life I would have after taking that, you know, just for preventative, in the event that there may not even be a chance that I’d… develop breast cancer.” Likewise, women wondered whether the gel posed a threat to others who touched it or touched a woman’s skin after she used the gel. For example, a participant questioned “Do you apply this with a bare hand? Then it’s on my hand and I’m doing dishes or cooking or putting a lunch together, you know, where does, how is that transferring?” They were also concerned about the impact of the gel on intimacy and on a woman’s ability to breastfeed. Women also noted concerns about cost and insurance coverage. One woman commented, “…I don’t know if I’m going to be committed to buying a pharmaceutical daily to prevent something that I may or may not get, you know, but if it’s once a year and it’s included in my health plan, and I don’t have to pay, you know, a whole bunch for it.” A number of other concerns were also mentioned, including interactions with other lotions or foods/beverages a desire to see data to support the gel’s efficacy.

## Discussion and Conclusions

Our data, from a highly educated group of healthy women at varying levels of perceived and calculated breast cancer risk, demonstrate surprisingly low awareness of breast cancer preventive medications. This was despite their high educational attainment, adherence to screening and motivation to seek information about breast cancer detection and prevention —100 % of the women over 40 had a screening mammogram and 100 % of those younger than 40 had attended an appointment in the comprehensive breast center to have a clinical breast exam and to learn more about their own risk. We have evaluated, in a rigorous and structured manner, the preference of this group for the mode of delivery of preventive medications for breast cancer. When preventive medications were described to these women, 91 % of women expressed preference for a skin application over an oral form. There was no relationship between calculated or perceived breast cancer risk and preference for the route of medication delivery. The number of women who accurately estimated their own risk, 50 %, was higher than most previously published studies, which report frequent over-estimation of risk, especially among women of European ancestry [19–21]. However, 60 % of high-risk women in our sample (excluding women who were unsure of their risk) correctly perceived themselves to be high risk. This proportion is much higher than findings from a recent publication in which as few as 18 % of women calculated to be at high risk using the risk assessment tools correctly perceived themselves to be at increased risk for developing the disease [22]. Our subjects’ high level of education and adherence to screening recommendations could account for the high number of women who correctly assessed their risk.

Our findings also highlight information women would want to know about skin applications of preventive medications, including their impact on day-to-day life, dosage, characteristics of the medications, side effects, and whether the topical medication posed a threat to others. Notably, many of the women’s questions about the gel, such as dosage and application, could be addressed via a brief discussion with a physician or via product packaging. Women also were concerned about the efficacy of a new medication delivery method, cost, insurance coverage, and determination of the population that would benefit most from the medicine. The general consensus of this sample of women was that if these concerns could be adequately addressed then women would be more likely to accept a medication for chemoprevention. Thus, a skin application with limited systemic effects has the potential to gain acceptance among at risk women.

Our study has several limitations. The women’s preferences were based on hypothetical situations—women were not actually confronted with the option of taking a pill or gel for breast cancer prevention. Moreover, our results were based on a small sample of mostly well-educated women from one large city. Each focus group expressed many of the same concerns; thus we feel confident that we have captured the most salient concerns for these women. Only four women in our sample were under the age of 35, the group where fertility concerns are an important issue, and could be an influential factor in decisions regarding preventive therapy for breast cancer. Future work should examine how such concerns might impact the willingness to take preventive medications. Additionally, work with different populations of women may reveal other concerns about preventive medications and LTT. Our sample represents women who may be most likely to take a preventive medication because of their high risk status and overall attention to their breast health, as evidenced by their regular mammogram screening and use of clinical breast exams. In addition, our sample, which has a mean age of 48, provides insights into the views of younger women towards preventive medicine; this is particularly important as this is the group least likely to accept preventive medications [4]. These women, with long potential life expectancies, stand to benefit most from preventive measures and are the target population for chemopreventive agents [23].

Additional studies of the long-term efficacy of LTT are needed and will increase women’s confidence in these novel medications. In addition, increasing public awareness of chemopreventive medications for breast cancer should be a goal of public health initiatives to encourage acceptance of preventive medications. Notably, the women in our study were very interested learning more about the gel, including when it would be available. Future work should also focus on establishing the side effect reduction of topical 4-OHT over oral tamoxifen. Because breast cancer chemoprevention requires a daily, long-term commitment from women, it is not surprising that many women refuse or stop taking the medication due to side effects and daily burden [13, 24]. Even more worrisome, women already facing the severity of a cancer diagnosis are discontinuing these medicines before they achieve full benefit due to these same concerns. The rates of adherence to oral adjuvant endocrine therapy have been reported to be as low as 12–59 % in women with cancer, and are understandably even lower in women taking the medicine for prevention [4, 7, 8]. Attention must be paid to improving acceptance and adherence to these drugs. For the healthy high risk and DCIS populations, LTT offers a promising approach to meeting this objective, which may in turn reduce the incidence and burden of breast cancer.

As additional, larger studies of LTT are designed, it will be important to address issues related to non-compliance that have been identified in the therapeutic setting and may apply to the prevention setting, regardless of the mode of delivery. These include the presence of comorbidities, negative mood prior to the initiation of endocrine therapy, and greater perceived bother associated with multiple symptoms [25–27]. Similarly, psychosocial characteristics were identified in an epidemiologic review as the most robust correlates of both non-adherence to therapy (i.e., patient– oncologist relationship quality, perceived need for endocrine therapy, endocrine therapy-related negative emotions and depressive symptoms) [8]. Low perceived financial status which has been shown to apply to populations with both high and low incomes [8, 27, 28]. Our group similarly voiced concerns over side effects of preventive medications and did inquire about the cost of a new medication and whether their insurance provider would cover it. This will be an important parameter; particularly since transdermal formulations of many drugs are often priced higher than oral formulations. Comparing the concerns discussed in our focus groups to those highlighted in previous studies illustrates the fact that there are several reasons for low adherence to oral tamoxifen. Addressing the concerns of at risk women and removing the barriers they have identified should lead to a boost in acceptance and compliance with preventive medicines.

## Ethics, consent and permissions

The study obtained approval from the Institutional Review Board of Northwestern University. The IRB approval number is STU00079604. Although no identifying information is used, the study participants provided informed consent in the form of verbal agreement at the beginning of each focus group interview.
